# Frequency-Specific Resting Connectome in Bipolar Disorder: An MEG Study

**DOI:** 10.3389/fpsyt.2020.00597

**Published:** 2020-06-24

**Authors:** Masakazu Sunaga, Yuichi Takei, Yutaka Kato, Minami Tagawa, Tomohiro Suto, Naruhito Hironaga, Takefumi Ohki, Yumiko Takahashi, Kazuyuki Fujihara, Noriko Sakurai, Koichi Ujita, Yoshito Tsushima, Masato Fukuda

**Affiliations:** ^1^Department of Psychiatry and Neuroscience, Gunma University Graduate School of Medicine, Maebashi, Japan; ^2^Tsutsuji Mental Hospital, Tatebayashi, Japan; ^3^Gunma Prefectural Psychiatric Medical Center, Isesaki, Japan; ^4^Brain Center, Faculty of Medicine, Kyushu University, Fukuoka, Japan; ^5^Department of Neurosurgery, Osaka University Medical School, Suita, Japan; ^6^Department of Genetic and Behavioral Neuroscience, Gunma University Graduate School of Medicine, Maebashi, Japan; ^7^Department of Diagnostic Radiology and Nuclear Medicine, Gunma University Graduate School of Medicine, Maebashi, Japan

**Keywords:** magnetoencephalography, bipolar disorder, resting-state network, graph theory, salience network, limbic network, depressive symptoms

## Abstract

**Background:**

Bipolar disorder (BD) is a serious psychiatric disorder that is associated with a high suicide rate, and for which no clinical biomarker has yet been identified. To address this issue, we investigated the use of magnetoencephalography (MEG) as a new prospective tool. MEG has been used to evaluate frequency-specific connectivity between brain regions; however, no previous study has investigated the frequency-specific resting-state connectome in patients with BD. This resting-state MEG study explored the oscillatory representations of clinical symptoms of BD *via* graph analysis.

**Methods:**

In this prospective case-control study, 17 patients with BD and 22 healthy controls (HCs) underwent resting-state MEG and evaluations for depressive and manic symptoms. After estimating the source current distribution, orthogonalized envelope correlations between multiple brain regions were evaluated for each frequency band. We separated regions-of-interest into seven left and right network modules, including the frontoparietal network (FPN), limbic network (LM), salience network (SAL), and default mode network (DMN), to compare the intra- and inter-community edges between the two groups.

**Results:**

In the BD group, we found significantly increased inter-community edges of the right LM–right DMN at the gamma band, and decreased inter-community edges of the right SAL–right FPN at the delta band and the left SAL–right SAL at the theta band. Intra-community edges in the left LM at the high beta band were significantly higher in the BD group than in the HC group. The number of connections in the left LM at the high beta band showed positive correlations with the subjective and objective depressive symptoms in the BD group.

**Conclusion:**

We introduced graph theory into resting-state MEG studies to investigate the functional connectivity in patients with BD. To the best of our knowledge, this is a novel approach that may be beneficial in the diagnosis of BD. This study describes the spontaneous oscillatory brain networks that compensate for the time-domain issues associated with functional magnetic resonance imaging. These findings suggest that the connectivity of the LM at the beta band may be a good objective biological biomarker of the depressive symptoms associated with BD.

## Introduction

Bipolar disorder (BD) affects up to 60 million individuals globally (1). BD can lead to cognitive and functional impairments (2) and is associated with a high mortality rate, particularly as a result of suicide (3). Although suicide attempts can be triggered by stressful life events such as bereavement (4) and biological abnormalities (5), the presence of a psychiatric disorder is the most consistent risk factor for suicidal behavior (6). Therefore, it is important to diagnose BD in the early clinical stage. However, it is difficult to diagnose BD quickly and accurately in clinical practice because its onset is most commonly characterized by a depressive episode, which often resembles unipolar depression (7). The efficacy of pharmacological interventions in the treatment of BD depends on an accurate and early diagnosis (8); however, a diagnostic biomarker has yet to be identified (9). Accordingly, for the accurate and timely diagnosis of BD, there is an urgent need to establish objective clinical biomarkers that are based on a better understanding of BD neuropathology. Studies investigating the resting-state functional connectivity have revealed intrinsic properties of brain disorders (10–13) and are expected to help identify such biomarkers.

Until recently, most studies investigating the functional connectivity in psychiatric patients have used functional magnetic resonance imaging (fMRI) (14). Although fMRI has a high spatial resolution, it measures an indirect neural activity and can only investigate slow oscillations in brain activity (< 0.1 Hz) (15). Compared to fMRI, a major advantage of magnetoencephalography (MEG) is its high temporal and spatial resolution, which enables synchronizations of the local and long-range neural activities to be assessed (16). In addition, the source reconstruction approach using MEG provides valuable spatial information that characterizes neural network modulations (14). Although MEG has many advantages, to our knowledge, there have been no studies of resting-state MEG for evaluating functional connectivity in patients with BD.

Unfortunately, a lack of established methodologies has prevented its widespread use for brain resting-state connectivity studies. In recent years, however, two British groups have developed methodological approaches to evaluate the frequency-specific resting-state connectivity using MEG (17, 18). These advances have enabled the investigation of frequency-specific alterations of resting-state networks *via* graph theory in autism (19) and age-related changes in these networks (20).

Simultaneously, graph theory analysis has been applied to explore brain network connectivity in psychiatric patients (21–25). Graphs are defined as sets of nodes and edges that represent system components and their mutual relationships (26). Using graph theory, brain networks can be represented as graphs that consist of nodes (which correspond to brain regions) and edges (which represent their connections) (27, 28). Therefore, graph theory is ideally suited for investigating complex networks such as human brain networks (26) and may be a potential tool for investigating the network characteristics of psychiatric disorders that alter connectivity within brain networks (29).

The main objective of this study was to investigate the resting-state frequency-specific functional connectivity to glean deeper insights into the pathophysiology of BD and help identify objective clinical biomarkers for the disorder. Here, we describe a novel MEG analysis that explores the resting-state functional connectivity using graph analysis in patients with BD. We hypothesized that resting-state MEG studies would provide important information about the spontaneous oscillatory brain networks, thereby representing an advantage over the sole use of fMRI-based resting-state investigations. We believed that this approach could also help identify clinical objective biomarkers for psychiatric disorders. To achieve this goal, we explored oscillatory representations of subjective and objective depressive symptoms in patients with BD using graph analysis.

## Materials and Methods

### Participants

Seventeen patients with BD and 22 healthy controls (HCs) participated in this prospective case-control study. All patients with BD were recruited from the Gunma University Hospital in Gunma Prefecture, Japan (**Table 1**). All enrolled participants were right-handed, as assessed using the Edinburgh Handedness Inventory (30). To diagnose BD and to exclude participants with a history of psychiatric disorders from the HCs, we administered the Structured Clinical Interview of the Diagnostic and Statistical Manual of Mental Disorders, Fourth Edition for Axis I (31) and Axis II Disorders (32). Subjective and objective depressive and manic symptoms were assessed using the Beck Depression Inventory—Second Edition (BDI-II), Altman Self-Rating Mania (ASRM) scale, 17-item Hamilton Depression Rating (HAMD-17) scale, and Young Mania Rating Scale (YMRS). Patients older than 60 years were excluded to rule out the possible interference of additional pathophysiological factors, such as aging and cerebrovascular changes. Most of the patients with BD (14/17) were using medications, including antipsychotics, mood stabilizers, antidepressants, anxiolytics, hypnotics, and/or antiparkinsonian drugs. The equivalent doses of antidepressants (imipramine equivalent dose in mg/day), antipsychotics (chlorpromazine equivalent dose in mg/day), anxiolytics (diazepam equivalent dose in mg/day), and hypnotics (flunitrazepam equivalent dose in mg/day) were calculated for each patient (33). HCs had no history of major psychiatric or physical illnesses and medication use. The exclusion criteria for both groups included a clear abnormality in brain magnetic resonance imaging (MRI) findings, neurological illness, traumatic brain injury with a known cognitive consequence or loss of consciousness for more than 5 min, substance use or addiction, and the presence of hearing or vision impairment. The study protocol was approved by the Ethics Committee of the Gunma University Graduate School of Medicine, Japan. All participants provided written informed consent before the study. This study was carried out in accordance with the latest version of the Declaration of Helsinki.

**Table 1:** Detailed demographics, clinical, and medication information of this study.

	HC (n = 22)		BD (n = 17)		*t* (χ^2^)	*p*
	M	F	M	F		
Sex	11	11	8	9	0.0198	0.888
	Mean	SD	Mean	SD		
Age (years)	44.5	5	47.1	6.1	-1.4643	0.152
JART score	110.2	9.1	111.4	11.5	-0.3467	0.731
GAF score	82.3	4.7	60.6	9.8	9.1539	0.000
BDI-II score	5.6	5.4	18.5	15.5	-3.6594	0.0008
ASRM score	3.7	2.6	3.5	4.2	0.1944	0.8469
HAMD-17 score	–		6.9	4.8	–	
YMRS score	–		0.5	1.5	–	
Antidepressants (mg/day)	–		56.2	97.9	–	
Antipsychotics (mg/day)	–		78.7	134.4	–	
Anxiolytics (mg/day)	–		19.9	32.9	–	
Hypnotics (mg/day)	–		0.5	1.1	–	
Lithium (mg/day)	–		164.7	247.3	–	
Lamotrigine (mg/day)	–		29.4	98.5	–	
Valproate (mg/day)	–		211.7	286.9	–	
Carbamazepine (mg/day)	–		82.3	187.8	–	

Antidepressants, imipramine equivalent dose; Antipsychotics, chlorpromazine equivalent dose; Anxiolytics, diazepam equivalent dose; Hypnotics, flunitrazepam equivalent dose; M, male; F, female; BD, patients with bipolar disorder; HC, healthy controls; JART, Japanese Adult Reading Test; GAF, Global Assessment of Functioning; BDI-II, Beck Depression Inventory–Second Edition; ASRM, Altman Self-Rating Mania Scale; HAMD-17, 17-item Hamilton Depression Rating Scale; YMRS, Young Mania Rating Scale.

### MEG Data Acquisition and Preprocessing

For each participant, we acquired the MEG data for 7 min. A crosshair was presented as a fixation point in front of the participants. The only instruction provided to participants was to keep their eyes open, to relax, and remain awake. To confirm the awareness level and evaluate the extent of sleepiness during MEG acquisition, we used the Stanford Sleepiness Scale (34).

During the experiment, participants sat in an upright position. The neuromagnetic signals were acquired inside a magnetically shielded room (JFE Mechanical Co., Tokyo, Japan) using a whole-head Elekta Neuromag Vector View system (Oy, Helsinki, Finland), which was composed of 306 sensors arranged as 102 triplets (each unit consisting of two orthogonal planar gradiometers and one magnetometer). First, the locations of three fiduciary points (the nasion, and left and right auricular points) defining the head frame coordinate system and a set of head surface points (approximately 150–200 points) (35), as well as those of four head position indicator coils, were digitized using an Isotrak three-dimensional digitizer (Polhemus™, Colchester, VT, USA). During acquisition, the sampling rate was 1000 Hz, and the signals were bandpass filtered between 0.1 and 200 Hz. All off-line analyses were conducted using continuous raw data.

The current source estimate was carried out as follows. For noise suppression, we applied the “oversampled temporal projection” method to improve the sensor noise levels (36). Subsequently, the data were spatially filtered using the signal space separation method (37, 38) with Maxfilter (Elekta, Stockholm, Sweden) to suppress the noise generated by sources outside the brain. A notch filter was used to eliminate noise from the power line (50 Hz) and its harmonics (100 and 150 Hz). We eliminated heartbeat- and eye movement-related artifacts from the raw data using independent component analysis (ICA). The ICA was applied to the MEG sensor signals, and the eye movement signals were isolated based on the hybrid approach of component discrimination using the time (signal waveform) and space (topology) (39).

### MRI

Brain MRI scans were acquired using a Siemens 3 T Trio scanner with a 12-channel head coil (Siemens, Erlangen, Munich, Germany) at the Gunma University Hospital and Josai Clinic (Philips Medical Systems, Best, Netherlands). High-resolution T1-weighted anatomical images were acquired using magnetization-prepared rapid acquisition with a gradient-echo sequence. Imaging parameters at the Gunma University Hospital were as follows: repetition time (TR) = 2000 ms, echo time (TE) = 2 ms, inversion time = 990 ms, flip angle = 9 degrees, field of view = 256 × 256 mm^2^, matrix size = 256 × 256, and voxel size = 1 × 1 × 1 mm^3^. Imaging parameters at the Josai Clinic were as follows: repetition time (TR) = 6.2 ms, echo time (TE) = 2.8 ms, flip angle = 9 degrees, field of view = 256 × 256 × 211 mm (anterior to posterior: AP × foot to head: FH × right to left: RL), matrix size = 256 × 256, and voxel size = 1 × 1 × 1 mm^3^. For anatomical segmentation and brain surface images, we first applied the FreeSurfer reconstruction process to the individual MRI data. Cortical reconstructions and parcellations for each participant were generated from T1-weighed MRI (40–42).

### MEG Source Imaging

For the MEG source localization analysis, we used the minimum norm estimates (MNE) suite and MNE python (43). We employed the boundary-element method, with a single compartment bound by the inner surface of the skull for the forward head modeling (44). The source current distribution was estimated using the noise-normalized dynamic statistical parameter mapping (dSPM) method (45, 46). The noise covariance matrix was computed from the empty room acquisition data. To reduce the bias of the estimated source locations towards the superficial currents, we incorporated the depth-weighting parameter (depth = 0.8) by adjusting the source covariance matrix (47).

### Data Analysis Procedure for Resting-State Networks

**Figure 1:**
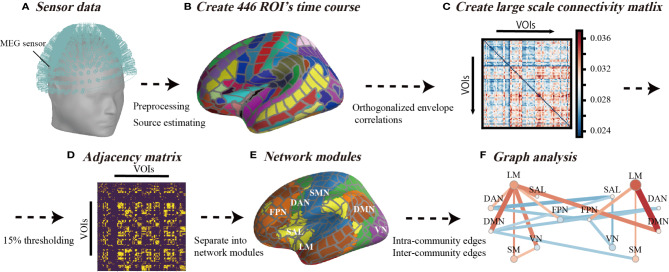
Schematic view of analysis flow. **(A)** Sensor data from 306-channel magnetoencephalography (MEG) and the application of preliminary filtering and noise elimination. **(B)** The source distribution image was estimated using minimum norm estimates, and source waveforms were extracted from 446 regions of interest. **(C)** Analysis flow of orthogonalized correlation. The correlation analysis of the power envelopes from the regions of interest source waveforms. **(D)** The threshold of the correlation matrix was held at the top 15% to ensure that the density or number of connections of the network was equal across all individuals. **(E)** A total of 446 regions of interest were classified for seven networks in each hemisphere. **(F)** The inter-community edges and intra-community edges were calculated from connections separated into seven networks.

Schematic images of the data analysis procedure used in this study are illustrated in **Figure 1**. We started with MEG sensor detection level analysis and then evaluated the source distribution as described in subsection *MEG Data Acquisition and Preprocessing* (**Figure 1A**). Subsequently, we imported the commonly employed cortex segmentation (Destrieux Atlas) from the FreeSurfer software, which automatically divided the cortex into 150 regions (48). After discarding the “Unknown” regions, the regions were further subdivided into a total of 446 cortical labels (20) (**Figure 1B**), such that each label covered almost identical areas through all the individual and standard brains. We employed this high-resolution parcellation because the cortical surface was convoluted, and the estimated source data were averaged across a large label, which crosses multiple sulci and gyri and possibly results in signal cancellation across the parcel (20). We first applied the above process to the standard brain (Montreal National Institute 305; provided by FreeSurfer software) (49), and then projected this onto each of the individual brains. Source leakage correction was essential for the evaluation of long-range correlations with MEG (50) and was determined in this study as described below.

As shown in **Figure 1C**, we applied the orthogonal correlations to compute the connectivity metrics involved in handling source leakages using the “envelope_correlation” function in MNE (17, 20) at delta (1–4 Hz), theta (4–8 Hz), alpha (8–12 Hz), low beta (13–20 Hz), high beta (20–30 Hz), and gamma (30–80 Hz) bands (17). The “envelope_correlation” obtains the correlation values according to the following calculation flow. We started with *x*(*t*) and *y*(*t*) as the extracted source waveforms from specific ROI, where *t* denotes time stamps. First, the extracted source waveforms *x*(*t*) and *y*(*t*) were bandpass filtered at each frequency band and acquired *x*(*t,f*) and *y*(*t,f*). For each step-frequency band, the complex signal, *x*(*t,f*) and *y*(*t,f*), was generated with the Hilbert transform from *x*(*t,f*) and *y*(*t,f*). The connectivity between the two complex signals *x*(*t,f*) and *y*(*t,f*) was calculated by orthogonalizing one signal with respect to the other *Y*_⊥*X*_ (*t*,*f*).

(1) $$Y_{⊥X}\;\left(t,f\right)=Im\left(Y\left(t,f\right)\frac{X{\left(t,f\right)}^{*}}{\left|X\left(t,f\right)\right|}\right)$$

The superscript * denotes the conjecture of the complex value, and “*Im*” denotes the imaginary part of the complex value. The power envelopes *Env*_*X*_(*t*,*f*) were subsequently determined by computation of the absolute value of *X*(*t*,*f*), as follows:

(2) $$Env_{X}\left(\text{t},f\right)=\left|X\left(t,f\right)\right|=\sqrt{X\left(t,f\right)X{\left(t,f\right)}^{*}}$$

Similarly, $Env_{Y_{⊥X}}\left(t,f\right)$ was obtained from *Y*_⊥*X*_ (*t*,*f*). We calculated the linear correlation between $Env_{Y_{⊥X}}\left(t,f\right)$ and $Env_{Y_{⊥X}}\left(t,f\right)$. Given the slow time course of these envelopes, and to ensure that enough independent samples were available in the correlation window (17), we calculated the orthogonal correlation using an overlapping sliding window of 30 s with a stride of 1/6 of the window size. Finally, we calculated the median values of sliding correlation values and applied the graph-theoretical analysis to these median correlation values (**Figure 1C**) as described below.

### Graph Analysis

We used the graph-theoretical measures (intra-community edges and inter-community edges) to quantify the basic property of resting-state networks (RSNs). We used the connectivity matrix that contained the orthogonal correlations between all N × N node pairs for each frequency band and acquired a connectivity array with the dimensions of N_Nodes_ × N_Nodes_ × N_Bands_ for each subject. Thresholding and binarizing the connectivity matrices resulted in adjacency matrix A (**Figure 1D**). We used a threshold proportional scheme to retain a given proportion of the strongest connectivity matrix entries in the connectivity matrix. As there is no rationale for using a cost threshold, we compared the graph network properties for a wide range of costs and used a thresholding range of 5% to 30% at increments of 5%. There was no significant difference among thresholding values (**Figure S1**), therefore, we set a threshold at the top 15% to ensure that the number of network connections (14,851 connections among all regions of interest) was the same across all individuals.

**Figure 2:**
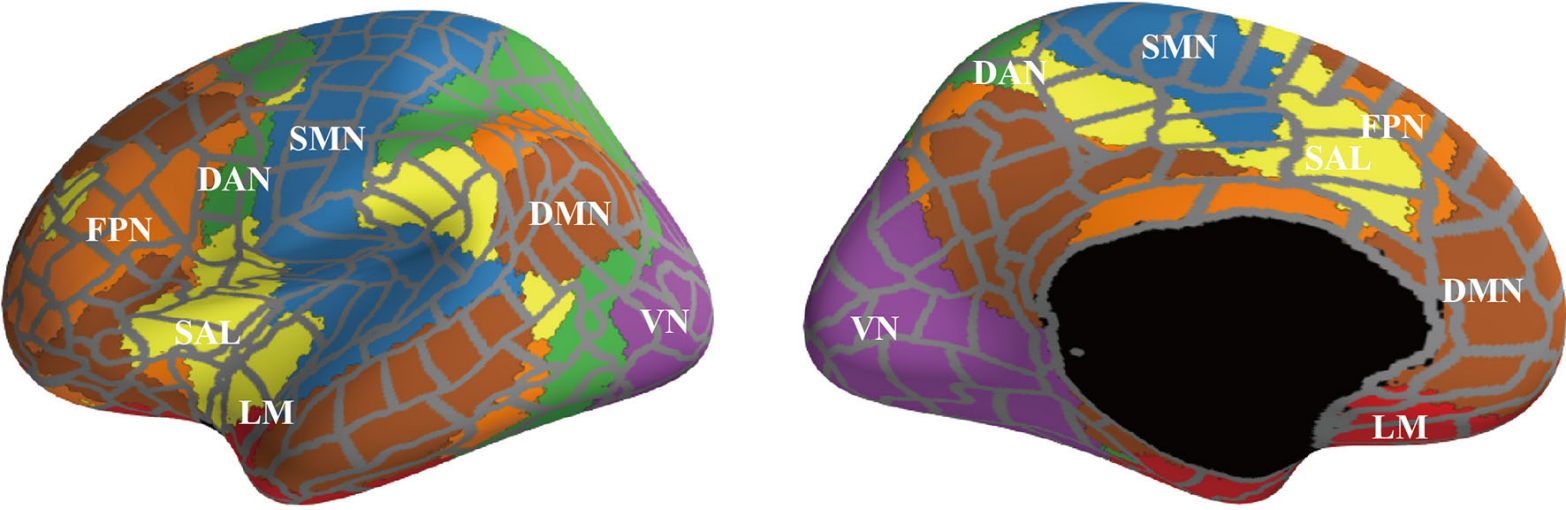
The surface areas of each network module. We separated regions of interest into seven left and right network modules [FPN, frontoparietal network (orange); LM, limbic network (light green); SAL, salience network (yellow); DAN, dorsal attention network (green); DMN, default mode network (brown); SMN, somatomotor network (sky blue); and VN, visual network (deep purple)], according to the network template.

We investigated the changes of RSNs in BD from the existing network understanding framework provided by previous fMRI studies. According to the network template, we separated network modules into seven left and right network modules, including the frontoparietal network (FPN), limbic network (LM), salience network (SAL), dorsal attention network (DAN), default mode network (DMN), somatomotor network (SMN), and visual network (VN) (51) (**Figures 1E** and **2**). In this way, we investigated differences in the connection patterns of intra- and inter-networks community between BD and HC groups. These network templates do not contain sub-cortical regions. We subsequently subdivided all edges into intra-community edges, which connect regions within each network, and inter-community edges, which connect inter-network regions, and measured the number of intra-community and inter-community edges for each frequency band (**Figure 1F**).

We compared the numbers of inter-community and intra-community edges for each frequency band between the HC and BD groups using an independent *t*-test in SciPy (52). Group differences were considered to be statistically significant at a *p*-value of less than 5%. We examined the relationship between significantly different edges (*p*-value less than 1% significance level) in patients with BD and clinical measures (the HAMD-17, BDI-II, ASRMS, and YMRS) using Spearman's rank correlation analysis. The correlation values were determined at a significance level less than 5%.

## Results

### Sample Characteristics

**Table 1** summarizes the demographic characteristics of the sample. Age and sex ratios were not significantly different between the two groups (*t* = -1.4643, *p* = 0.152; chi-square = 0.0198, *p* = 0.888). At the time of the study, the patients with BD were euthymic or depressive as indicated by their HAMD-17 and YMRS scores.

### Inter- and Intra-Community Edges

**Figure 3:**
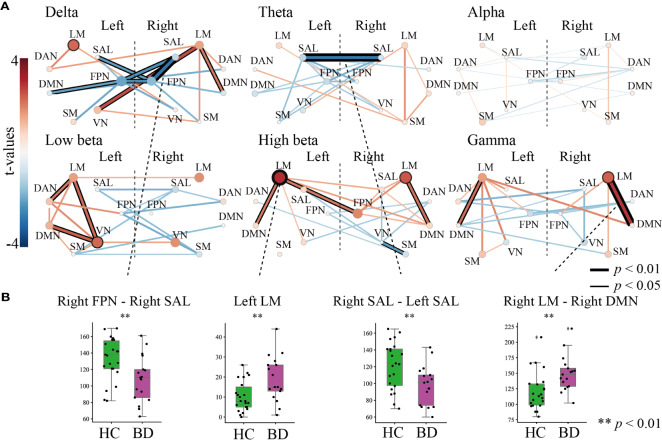
The results of the graph theory analysis for inter- and intra-community edges. **(A)** Graph theory was applied to six frequency band ranges. The left-most bar indicates the color scale of the t-value. All figures in **(A)** indicate *t*-values of results that compared inter-community edges (line thickness and color) and intra-community edges (circle size and color) between healthy controls and patients with bipolar disorder (BD) at each frequency band. Thicker and deeper lines indicate higher inter-community edges, and larger circles indicate higher intra-community edges in patients with BD. If the *p*-value of the inter- or the intra-community edges was below the threshold for significance, the circles or lines are bordered by a thick line *(p <* 0.01) or thin line *(p <* 0.05). **(B)** The results of further statistical analysis for stage **(A)**. The green boxplot shows the results from the HC group, and the purple boxplot shows the results from the BD group. Each vertical boxplot bar indicates the standard error.

**Figure 3** shows the results of the graph theory analysis, in which the number of inter- and intra-community edges were measured. **Figure 3A** depicts the summarized degrees of inter- and intra-community edges in six frequency band ranges (delta, theta, alpha, low beta, high beta, and gamma), including the plot box bars at a significance level < 1% (**Figure 3B**). We compared the number of edges between HC and BD using an independent *t*-test (**Figure 3A**, left).

In the delta band range, we found lower inter-community edges among the FPN and other network modules in the right FPN–right SAL (*t* = -2.808, *p* = 0.007), right FPN–left SAL (*t* =-2.079, *p* = 0.046), right FPN–left FPN (*t* = -2.423, *p* = 0.020), left FPN–left DMN (*t* = -2.043, *p* = 0.048), and left FPN–right SAL (*t* = -2.230, *p* = 0.027). The right LM showed higher inter-community levels involving three domains on the right LM–left VN (*t* = 2.621, *p* = 0.013), right LM–right DMN (*t* = 2.038, *p* = 0.049), and intra-community edges on the left LM (*t* = 2.261, *p* = 0.0300) (**Figure 3A**, upper left). In the theta band range, a decreased connectivity between the left and right SAL (*t* = -2.722, *p* = 0.009) was found in the BD group (**Figure 3A**, upper middle). Interestingly, almost all inter- and intra-community edges in the alpha band ranges were quite similar in both groups (**Figure 3A**, upper right). In the low beta band range, positive higher community edges were mainly increased in the left hemisphere, and inter-community edges were higher between the left VN–left LM (*t* = 2.281, *p* = 0.0284), left VN–left DMN (*t* = 2.391, *p* = 0.022), and left DAN–left LM (*t* = 2.100, *p* = 0.043), and higher intra-community edges on the left VN (*t* = 2.273, *p* = 0.029) (**Figure 3A**, lower left). We paid special attention to the results in the high beta band range and found increased community edges mainly in the LM. Higher inter-community edges were observed, as expected, in the left LM–right FPN (*t* = 2.094, *p* = 0.0431), left LM–left DMN (*t* = 2.038, *p* = 0.049), and right LM–right DMN (*t* = 2.201, *p* = 0.034), and higher intra-community edges on the left LM (*t* = 2.981, *p* = 0.005) and left RM (*t* = 2.480, *p* = 0.018) (**Figure 3A**, lower middle). Interestingly, the gamma band range also demonstrated higher inter-community edges in both the left and right LMs in this band range, especially for the right LM–right DMN (*t* = 2.899, *p* = 0.006) and left LM–left DMN (*t* = 2.205, *p* = 0.034), and higher intra-community edges on the right LM (*t* = 2.356, *p* = 0.024) (**Figure 3A**, lower right).

To summarize, among the six frequency band ranges, the LM exhibited higher community edges irrespective of the hemispheric differences, while the SAL and FPN exhibited a lower community edge among lower frequency band ranges. In particular, the number of inter- and intra-community edges were significantly different between the following groups at each frequency band (all, *p* < 0.01): the right FPN–right SAL at the delta, left SAL–right SAL at the theta (HC > BD), left LM at high beta, and right LM–right DMN at gamma (HC < BD) (**Figure 3B**).

### Relationship Between Characteristics of Graph Indices and Clinical Symptoms

**Figure 4:**
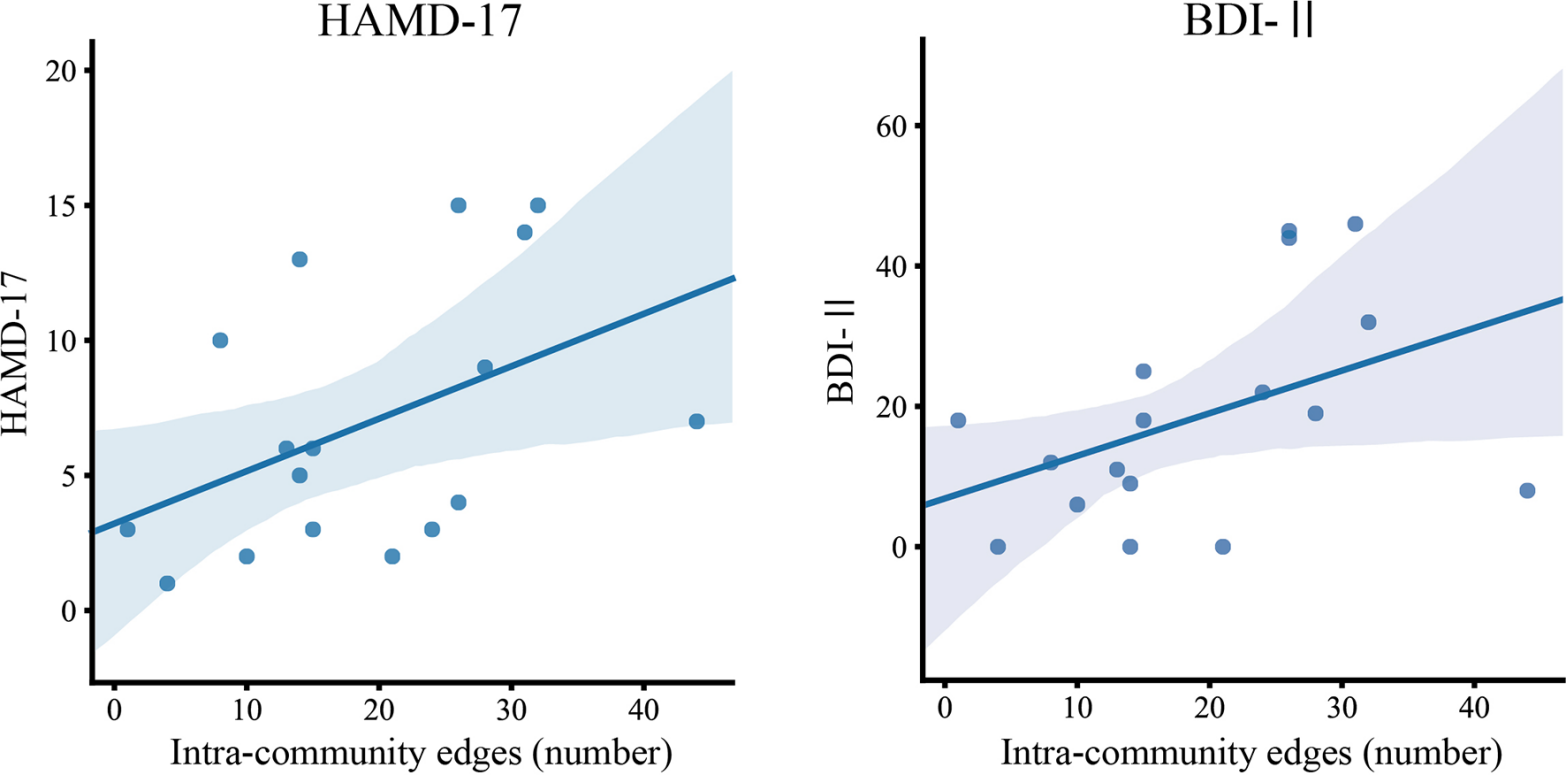
The relationship between intra-community edges and depressive symptoms. The scatter plot of the number of intra-community edges in the left limbic network at the high beta band, and the scores of the Beck Depression Inventory–Second Edition (BDI-II) and 17-item Hamilton Depression Rating Scale (HAMD) in patients with BD. Color-matched transparent areas represent the standard errors of the mean.

We further evaluated the clinical interpretations and relationships *via* commonly used approaches in psychiatric diagnosis. A Spearman's rank correlation analysis was used to assess the relationship between significantly different graph indices (inter- and intra-community edges) and clinical measures (BDI-II, HAMD-17, ASRM, and YMRS scores). **Figure 4** shows the relationship between graph indices and depressive symptoms. The Spearman's rank correlation analysis showed that the number of intra-community edges in the left LM at the high beta band was positively correlated with the HAMD-17 (*r* = 0.519, *p* = 0.032; **Figure 4**, left) and BDI-II (*r* = 0.522, *p* = 0.031) scores within the BD group (**Figure 4**, right). In contrast, age, drug dosages, and the JART, GAF, ASRM, and YMRS scores had no significant relationship with the graph indices.

## Discussion

In this study, we performed an analysis using graph theory and observed frequency-specific alterations of RSNs in patients with BD compared to HCs. We observed higher LM community edges at higher frequency band ranges, especially inter-community edges of the right LM–right DMN at the gamma band, and intra-community edges of the left LM at the high beta band, in the BD group. We also observed decreased inter-community edges at lower frequency band ranges, especially in those of the right SAL–right FPN at the delta band and the left SAL–right SAL at the theta band, in the BD group (**Figure 3**). The increase in the number of connections in the left LM at the high beta band was positively correlated with subjective and objective depressive symptoms in the BD group (**Figure 4**).

Several fMRI studies investigating the resting-state functional connectivity in the BD population have shown altered connectivity between parts of the DMN and the limbic system (14, 22, 53–58). The hyper-connectivity between the right LM and the right DMN of the BD group in the present study supports these previous findings and may indicate that alterations in resting-state functional connectivity between the LM and DMN are the essential elements associated with BD. The LM deals with emotional regulation/processing and memory, and previous studies have reported that patients with BD showed increased functional connectivity in the mesoparalimbic network (59, 60). The DMN is thought to be involved in self-referential activities such as inner speech, mental images, remembering past events, and planning future events (61, 62). The higher inter-community edges of LM–DMN suggest that self-referential activity tends to be affected by emotional activity, and we speculate that this tendency induces ruminative thoughts in patients with BD.

We also observed attenuation of the number of inter-community edges of the SAL–FPN at the delta band (**Figure 3A**, top left) and left SAL–right SAL at the theta band (**Figure 3A**, top middle) in the BD group. A previous study also reported a significantly altered functional connectivity between the bilateral dorsal anterior insula and the left inferior parietal lobe in patients with BD (63). The SAL has been implicated in playing an important role in switching between the central executive network and DMN (64). Furthermore, the SAL activates the central executive network to manipulate information stored in working memory, while suppressing the DMN to keep attention focused on task-relevant goals. The hypoconnectivity between the SAL-FPN and left SAL-right SAL may contribute to the difficulty in maintaining a cognitive set and to the focusing of attention on task-relevant goals in patients with BD.

Remarkably, we found that the number of connections in the left LM at the high beta band (**Figure 3A**, lower middle) had positive correlations with the subjective and objective depressive symptoms in the BD group. This finding is consistent with previous reports that alterations in connectivity in the beta band are associated with depressive symptoms in major depressive disorder (65, 66), which demonstrates that excessive connectivity of the LM in the beta band is associated with depressive symptoms. Salvadore et al. investigated the patterns of the anterior cingulate cortex (ACC) and amygdala activity in patients undergoing antidepressant treatment with ketamine using the task of fearful faces (65) and found that pretreatment amygdala activity was negatively correlated with changes in depressive symptoms. These results suggest that LM activity increases with an increase in the severity of depressive symptoms and that the connectivity of the LM in the beta band may be a good objective biological biomarker of the depressive symptoms.

A resting-state EEG study in a BD population found frequency-specific alterations of the network topologies (67). Although no resting-state MEG study investigating functional connectivity has been reported so far, frequency-specific inter-community edges, including the SAL change in the delta and theta bands in patients with BD, were clearly demonstrated in our study. These differences in inter-community edges in the SAL were observed as changes in the low-frequency bands such as delta and theta. A recent study reported that greater theta band connectivity between the right ACC and right anterior insula, a key region within the SAL, predicted a greater reduction in the severity of depression (68). Altered networks at the theta and delta bands have been reported to be associated with pain control and auditory hyperacusis (69, 70). Parker reported that patients with BD exhibited various suprasensory changes experienced during manic/hypomanic “high” states, which generally attenuate or disappear during depressive and euthymic periods (71). Network alternations at the theta band in the SAL may be related to the altered sensory perception of BD. Furthermore, patients with BD experience significantly increased levels of pain, particularly chronic pain and migraine. Difficulty in controlling pain in patients with BD may be related to a slow change in the oscillatory network in the SAL. In future studies, we aim to investigate the relationship between symptom formation and frequency-specific networks in BD.

## Limitations

Unfortunately, statistical analysis with a multiple comparison correction was not possible due to the insufficient number of subjects. Patients with BD in a hypomanic or manic state were not included; therefore, an investigation of the relationship between the manic symptoms and the connectome was not performed. Accordingly, large-scale prospective studies using MEG measurements in patients with BD are needed.

## Conclusion

In conclusion, we observed frequency-specific alterations of RSNs in patients with BD *via* MEG analysis using graph theory. To the best of our knowledge, this is the first resting-state MEG study investigating functional connectivity using graph theory in a BD population. We show that frequency-specific alterations in the inter-community and intra-community edges in the BD group, and the intra-community edges in the left LM in the high beta band were significantly positively correlated with subjective and objective depressive symptoms in the BD group. We suggest that the connectivity of the LM in the beta band may be a good objective biological biomarker of depressive symptoms in patients with BD. Also, the higher inter-community edges of LM–DMN suggest that self-referential activity tends to be affected by emotional activity in patients with BD. Moreover, the hypoconnectivity between the SAL-FPN and left SAL-right SAL may contribute to the difficulty in maintaining a cognitive set and to the focusing of attention on task-relevant goals in the BD group, and may be related to the altered sensory perception of BD. Further studies based on MEG analysis using graph theory would help elucidate the frequency-specific alterations of RSNs in psychiatric patients and identify biological biomarkers for psychiatric disorders.

## Data Availability Statement

The raw data supporting the conclusions of this article will be made available by the authors, without undue reservation.

## Ethics Statement

The studies involving human participants were reviewed and approved by Ethics Committee of the Gunma University Graduate School of Medicine, Japan. The patients/participants provided their written informed consent to participate in this study.

## Author Contributions

MT, MF, and YuiT designed the study. MS wrote the initial draft of the manuscript. YK, MT, and NH contributed to the analysis and interpretation of data and assisted in the preparation of the manuscript. All authors contributed to the article and approved the submitted version.

## Funding

This work was supported by the Grants-in-Aid for Scientific Research from the Ministry of Education, Culture, Sports, Science, and Technology; Grant-in-Aid for Young Scientists (B) (No. 16K19748); Grant-in-Aid for Scientific Research (C) (No. 19K08038): Grant-in-Aid for Scientific Research on Innovative Areas (No. 16H06397); and Novartis Research Grant 2018 and 2019.

The authors declare that this study received funding from Novartis. The funder was not involved in the study design, collection, analysis, interpretation of data, the writing of this article, or the decision to submit it for publication.

## Conflict of Interest

The authors declare that the research was conducted in the absence of any commercial or financial relationships that could be construed as a potential conflict of interest.
