# Supplementary material for: Frequency-Specific Resting Connectome in Bipolar Disorder: An MEG Study
Source: Front Psychiatry. 2020 Jun 24;11:597. doi: 10.3389/fpsyt.2020.00597 (PMC7330711; doi:10.3389/fpsyt.2020.00597)
Supplement: Supplementary file 1 [file DataSheet_1.docx]

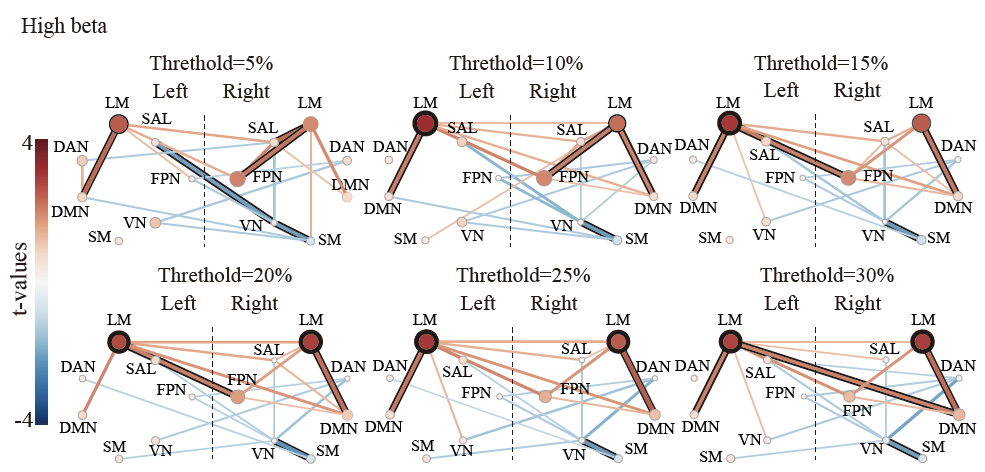


Figure S1. The results of the graph theory analysis for inter- and intra-community edges. Graph theory results at high beta band thresholding ranged from 5% to 30% at increments of 5%. The left-most bar indicates the color scale of the t-value. All figures indicate t-values of results that compared the inter-community edges (line thickness and color) and intra-community edges (circle size and color) between the healthy controls and patients with bipolar disorder (BD) at each frequency band. Thicker and deeper lines indicate higher inter-community edges and larger circles indicates higher intra-community edges in patients with BD. If the p-value of the inter- or intra-community edges was below the threshold for significance, the circle or line is bordered by a thick line (p < 0.01) or a thin line (p < 0.05).
